# Increasing Perspectival Obliqueness Increases the Leaning Tower Illusion

**DOI:** 10.1177/2041669518758778

**Published:** 2018-02-19

**Authors:** Giulia Parovel, Alan Costall

**Affiliations:** Department of Social, Political and Cognitive Sciences, University of Siena, Italy; Department of Psychology, University of Portsmouth, UK

**Keywords:** perception, perceptual organization, scene perception, spatial vision

## Abstract

The leaning tower illusion is a perceptual illusion in which two identical images of a tower photographed from below appear to diverge when juxtaposed. We manipulated the perceived obliqueness of the (upright) St Mark bell tower in Venice by modifying two parameters both related to the position of the camera with respect to the tower: (a) increasing the peripherality of the tower and (b) reducing the distance between the camera and the tower. The resulting images clearly show that the illusory leaning effect increases as a function of the obliqueness. Another crucial condition for the leaning tower effect must be that the twin images are perceived as parts of a unitary display: The illusion increases when the distance between the photos is progressively increased, but beyond a certain level of separation, the integration of the images should, of course, break down, and the illusion vanish.

Kingdom, Yoonessi, and Gheorghiu (2007) introduced an impressive new visual illusion, in which two identical images of the Leaning Tower of Pisa, when placed next to one another, produce the strong impression that the tower on the right leans more, as if photographed from a different angle (Figure 1).

**Figure 1.**
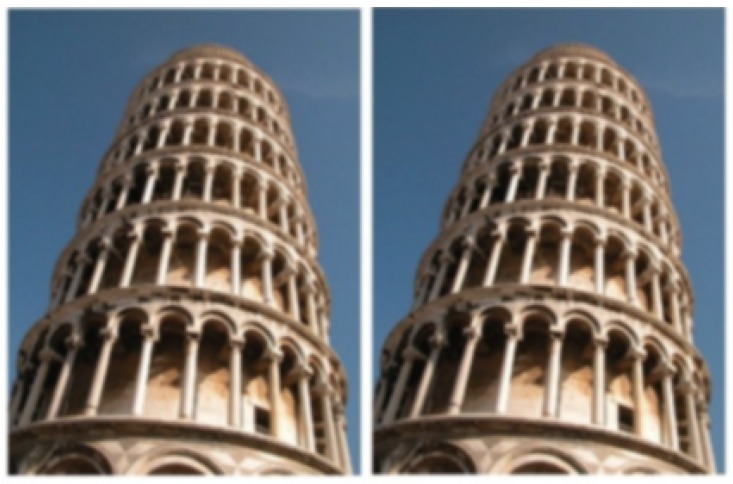
The leaning tower illusion.

According to these authors, the perceiver assumes that two side-by-side images are part of the same scene. In a unitary scene, they argued, when two towers are photographed from below, like the Petronas twin towers in Kuala Lumpur, they appear to converge as they recede in depth. In the case of the leaning tower (LT) illusion, if the corresponding outlines of a pair of physically identical receding objects are parallel in the two-dimensional projection, they cannot be really parallel but must be diverging as they recede from the viewing point (Kingdom et al., 2007).

Maniatis (2008) argued that the illusion was caused by orientation contrast. According to this author, the LT illusion is a variant of the Jastrow illusion—in which one of two identical shapes appears smaller than the other—but applied to perspective tilt. Yoonessi, Gheorghiu, and Kingdom (2008) replied providing further evidence for the perspective explanation. They observed that the illusion strongly depends on the elicitation of perspective depth and fails to take place with frontoparallel shapes, such as simple outline drawings of the towers. Moreover, Kingdom, Yoonessi, and Gheorghiu (2017) recently showed a pair of Pisa towers made from a photograph taken from a sufficient distance such that the tower does not appear to be a receding object, and there was no illusion.

What these approaches fail to address is a clear identification of the variables involved in determining the illusion, beyond saying that it depends in some ways on their perspectival character. But why do we not perceive a symmetrical divergence of the two towers and, instead, get the clear impression that the tower on the right is leaning more, as indeed Kingdom et al. (2007) themselves point out?

We agree with Kingdom et al. (2007) that a necessary condition for the LT effect must be that the twin images are perceived as parts of a unitary display. As a consequence of this, when the two photos are juxtaposed, the viewpoint of the observer no longer coincides with the station point of the original photo but is relocated in a new position halfway between the two. But at the same time, the foreshortening gradients of the structure of the two towers are identical.

As lateral foreshortening appears to be a sine qua non for the LT illusion, it seems reasonable to investigate whether the *degree* of tilt might affect the illusion. We therefore manipulated the obliqueness of the (upright) St Mark bell tower in Venice by modifying two parameters both related to the position of the camera station point with respect to the tower: (a) increasing the peripherality of the tower, that is, taking different pictures by rotating the camera in the horizontal plane and (b) reducing the distance between the camera station point and the tower, always keeping the tower at the side of the image (Figure 2(a) and (b)).^1^

**Figure 2.**
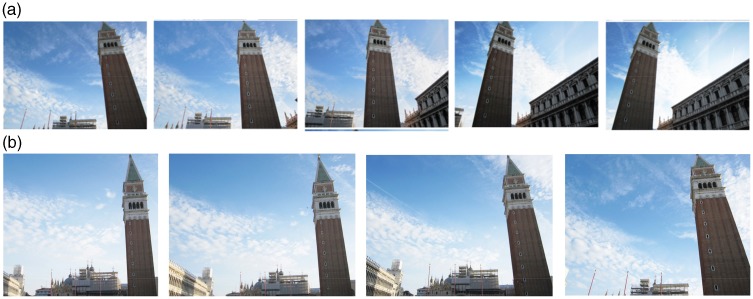
(a) The bell tower of St Mark’s Basilica in Venice photographed at different degrees of peripherality by rotating the camera in the horizontal plane. (b) The bell tower of St Mark’s Basilica photographed by placing the camera at different distances from it (keeping the tower at the side of the image).

To test the hypothesis about the effect of obliqueness in the LT illusion, we matched three pairs of identical towers for each condition (*peripherality* and *distance* of the camera station point) to obtain six stimulus pairs (see Figure 3(a) and (b)).

**Figure 3.**
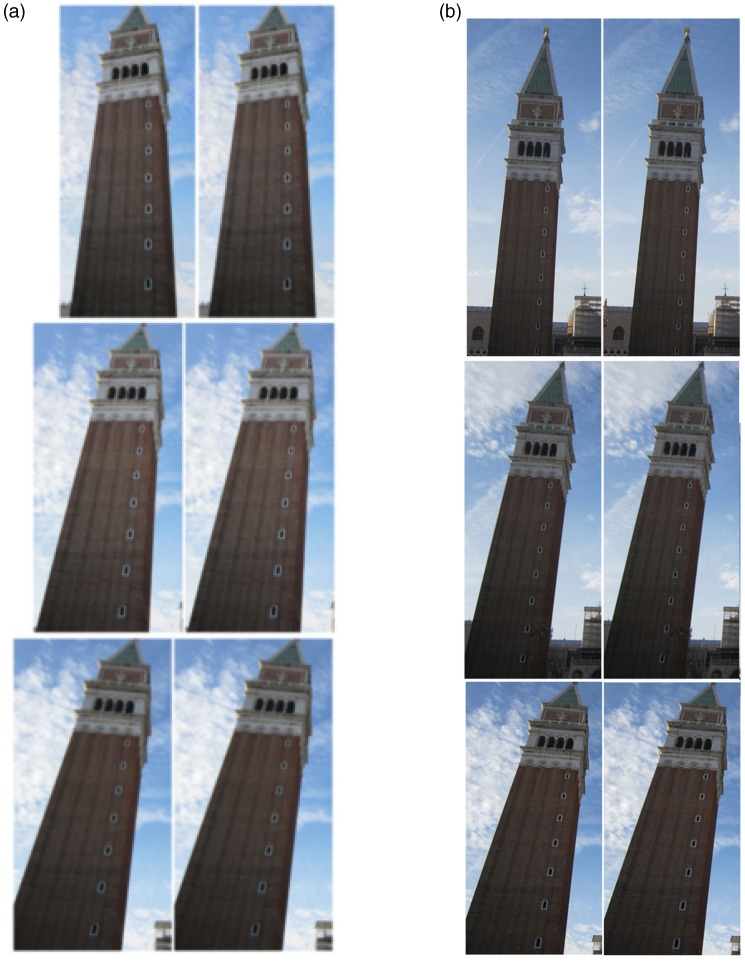
The LT illusion applied to St Mark’s bell tower with different degrees of obliqueness: (a) pictures taken at three different degrees of peripherality with respect to the camera station point; and (b) pictures taken at three different distances, that is, varying the slant of the tower, from the camera station point.

As Figure 3 clearly demonstrates, the magnitude of the illusion increases as a function of both the peripheral position and the distance between the camera station point and the tower. So we can infer that the best condition to obtain the LT illusion is to photograph a tower—and not necessarily a *leaning* tower—from below, keeping it at the periphery of the image, as much as possible.

Our demonstration shows that the obliqueness of the towers is a crucial factor in the LT illusion. As evident in the figure, an increase in the apparent divergence between the two towers correlates with the apparent increasing obliqueness of each tower. In fact, the greater the obliqueness, that is, the lateral foreshortening of the structural gradient, the greater the distance between each implicit tower's station point and the viewpoint of the observer of the unitary figure, and the internal conflict of the scene becomes stronger.

Another way to increase the internal conflict between the actual station point of the two identical images and the implicit station point of the visually unified figures may be obtained by somewhat increasing the distance between the photos as in Figure 4.

**Figure 4.**
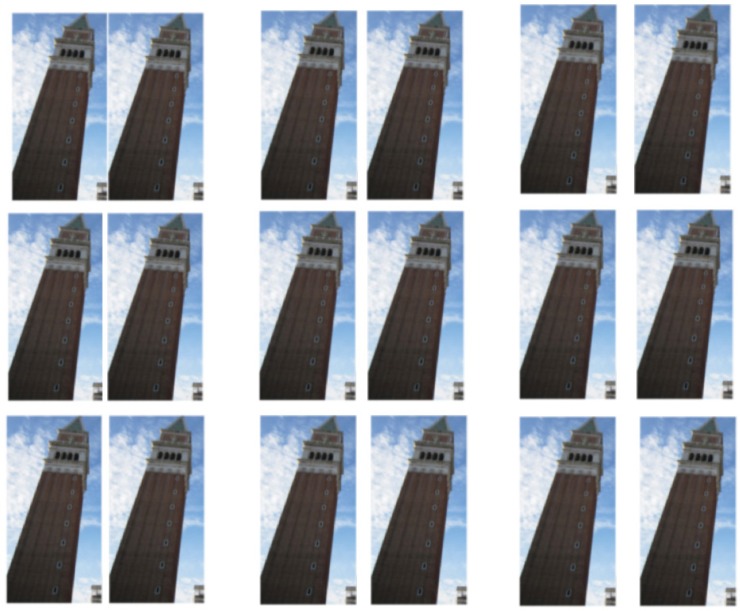
The distance between the photos is progressively increased, and the illusion increases too, until it breaks down as the photos become independent, as in Figure 5.

As we can see, consistent with our explanation about a conflict between the camera station points and the viewpoint of the observer of the image, increasing the distance between the photos the LT illusion also slightly increases. According to our argument, the effect should break down when the images are far apart. Figure 5 was our attempt to demonstrate this, but remarkably for some viewers the effect still occurs, despite the wide separation of the images and the fact that they cannot be taken in within one glance.

**Figure 5.**
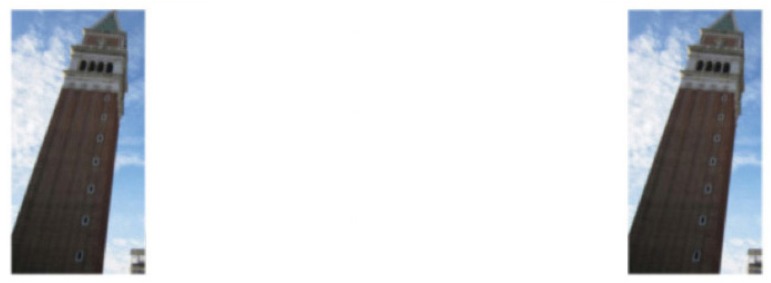
The distance between the towers is too great to group them together, but for some viewers the illusion still occurs.

## General Discussion

Finally, in what way does the LT illusion depend on the conflict between the foreshortening gradients of the structure of the two towers and the viewpoint of the observer of the double image?

Our hypothesis is that when the oblique orientation and the degree of foreshortening of the structure of the tower is consistent with its perspectival position in the scene, as defined from the central point of observation of the double image, the obliqueness is not attributed to the tower, but to the perspective itself (independently of whether the tower is *really* leaning or not). Instead, when the orientation of the tower and its structural gradient are inconsistent with its perspectival position in the double image, the obliqueness is then better attributed to the tower itself (see Arnheim, 1974; Zanforlin & Vallortigara, 1988). In other words, reconsidering the original version of the LT illusion, we would say that the perspective inconsistency between the foreshortening gradients of the towers and the viewpoint of the observer of the double image increases the leaning of the Tower of Pisa located in the right-hand part of the image, while consistent perspective attribution actually reduces the leaning of the Tower located on the left, or at least somewhat reduces its tilt.

Kingdom et al. (2007) state that “what the illusion reveals is not a failure of perspective per se, but the tendency of the visual system to treat two side-by-side images as if part of the same scene” (p. 475). We would extend this explanation by saying that the tendency of the visual system to treat two side-by-side images as if they were part of the same scene produces a reorganization of the whole visual field, defining a new point of observation from which the two towers are seen in the image by the viewer. Here, there is no “failure” of perspective, but perspectival conflicts themselves are resolved and specify a new meaning to the oblique orientations of each tower in the new image.

## Note

1 In other words, when we change the angle between the camera and the portrayed object, keeping the same center of rotation of the camera, we get a different orientation of the projection plane. It appears inclined along the horizontal axis, as in lateral *foreshortening* (Figure 2(a)), or along the vertical axis, as seen from below and receding toward the sky, as in Figure 2(b) (see Pirenne, 1991).
